# 

**Published:** 1880-05

**Authors:** 


| | PAGE. |
|---|---|
| **ORIGINAL COMMUNICATIONS:** | |
| Testimony of Medical Experts, by Hon. Charles Beckwith, | 421 |
| **CLINICAL REPORTS:** | |
| Epithelioma of the Cervix Uteri—Removal by Prof. James P. White, M. D. Reported by William D. Granger, M. D. | 428 |
| Hospital Notes—Sarcoma of Left Wrist. Reported by Dr. Frederick Peterson, House Physician at the Buffalo General Hospital—Service of Dr. C. C. F. Gay. | 430 |
| Compound Comminuted Fracture of the Cranium | 431 |
| **TRANSLATIONS:** | |
| Treatment of Club-foot by Excision of Bone from the Dorsum Pedis, by Prof. Koenig, in Gottingen. From the German by Herman Mynter, M. D. | 432 |
| **SELECTIONS:** | |
| Abortion and its Treatment: With Special Regard to the Injection of Warm Water. By J. Fletcher Horne, F. R. C. S. Edin. | 433 |
| General and Hygienic Treatment of Fibrinous Croup | 437 |
| Astringents in Chronic Conjunctivitis, by Dr. F. M. Wilson, Bridgeport, Conn. | 439 |
| Additional Evidence Concerning Particular Drugs | 440 |
| Tracheotomy for Diphtheritic Croup | 443 |
| Catarrh of the Bladder; Albumen in the Urine, by Sir Henry Thompson | 444 |
| The Presence of Alchohol in Animal Tissues | 447 |
| The Therapeutic Value of Pulsatilla, | 448 |
| Nitro-Glycerine | 449 |
| Treatment of Goitre by Chloride of Ammonium | 449 |
| Absorbing Power of Wounds | 450 |
| Toleration of Morphine | 450 |
| Production and Increase of Secretion of Milk by the Ricinus Communis | 451 |
| Tracheotomy for Croup | 451 |
| Night Medical Service | 452 |
| Gratuitous Services to Clergymen | 452 |
| The Thermometer in Trephining for Epilepsy | 452 |
| Chlorate of Potash in the Hæmorrhagic Diathesis, by A. Harkin, M. D., Belfast | 453 |
| Death from Nitrous Oxide, | 454 |
| A Shrewd Calculation on the Profits of Druggists | 454 |
| **SPECIAL COMMUNICATIONS:** | |
| Sale of Diplomas | 455 |
| Hydrate of Chloral | 459 |
| **EDITORIAL:** | |
| Clinical Reports | 460 |
| Buffalo Medical Association | 461 |
| Management of Insane Asylums | 462 |
| Fellows' Hypo Phosphites | 463 |
| **REVIEWS,** | 463 |

